# Association between eNOS 4b/a Polymorphism and the Risk of Diabetic Retinopathy in Type 2 Diabetes Mellitus: A Meta-Analysis

**DOI:** 10.1155/2014/549747

**Published:** 2014-05-08

**Authors:** Ze-jun Ma, Rui Chen, Hui-Zhu Ren, Xin Guo, Jun Guo, Li-ming Chen

**Affiliations:** Collaborative Innovation Center of Tianjin for Medical Epigenetics, Key Laboratory of Hormone and Development (Ministry of Health), Metabolic Disease Hospital & Tianjin Institute of Endocrinology, Tianjin Medical University, Tianjin 300070, China

## Abstract

Many studies have assessed the association between eNOS-4b/a polymorphism and the risk of diabetic retinopathy (DR) among type 2 diabetic subjects. However, the results are inconsistent. In order to derive a more precise estimation of the association, a meta-analysis was conducted. Fifteen studies with 3, 183 cases and 3, 410 controls were enrolled by searching the databases of Pubmed, Embase, China National Knowledge Infrastructure (CNKI), and Chinese Wanfang Database. Summary odds ratios (ORs) with 95% confidence intervals (CIs) were calculated. The main analysis indicated no significant association between eNOS-4b/a polymorphism and the risk of DR in overall population [allelic model: OR = 0.94 (0.79–1.11); additive model: OR = 0.91 (0.73–1.14); recessive model: OR = 1.01 (0.81–1.25); dominant model: OR = 0.91 (0.75–1.09)]. Subgroup analysis by ethnicity also indicated no significant association. In conclusion, the current meta-analysis did not observe any association between the polymorphism of eNOS 4b/a and the risk of DR among type 2 diabetic subjects. However, larger well-designed studies are required to confirm this finding.

## 1. Introduction

Diabetic retinopathy (DR) is one of the most common microvascular complications of diabetes mellitus (DM) and a leading cause of adult blindness worldwide [1, 2]. Although long duration of diabetes and poor control of glycemia have been considered as the major risk factors for the development of DR, accumulated evidences suggest a genetic influence on susceptibility to this complication [3]. A number of genes have been suggested as candidate genes of diabetic retinopathy, for example, methylenetetrahydrofolate reductase gene, endothelial nitric oxide synthase gene (eNOS), vascular endothelial growth factor gene, and so on [4–6].

The eNOS gene is located on chromosome 7q35-36 and includes 26 exons, spanning 21 kb. The polymorphism of eNOS 4b/a gene consists of the two alleles of eNOS 4a with 4 tandem 27-repeats and eNOS 4b with 5 repeats [7]. NO is produced through the oxidation of L-arginine by eNOS [8]. NO can regulate endothelial function and is an important factor in the maintenance of homeostasis. NO can contribute to vasodilatation, increase local blood flow, and decrease vascular resistance in ocular circulation. Studies on humans and animal models have suggested that eNOS plays an essential role in retinal vascular function and disequilibrium in its production can lead to the development of DR [9–11]. The presence of eNOS polymorphisms might contribute to a decreased eNOS activity and a reduced NO level and has been reported to be a potential factor in the pathogenesis and development of DR.

To date, many case-control studies have been carried out to investigate the relationship between eNOS-4b/a polymorphism and the risk of DR among type 2 diabetic subjects, but results of these studies were conflicting and inconclusive. Some studies observed that there was an association between eNOS-4b/a polymorphism and the risk of DR [4, 12], while some others suggested there was no significant association [13, 14]. To draw a more reliable conclusion, we performed a meta-analysis of all available studies dealing with the relationship between the eNOS-4b/a polymorphism and DR among type 2 diabetic subjects, including subgroup analyses based on different ethnicities.

## 2. Materials and Methods

### 2.1. Literature Search Strategy

We searched the literature databases including Pubmed, Embase, China National Knowledge Infrastructure (CNKI), and Chinese Wanfang Database. The last updated search was performed on November 20, 2013. The search used the following terms: “endothelial nitric oxide synthase or eNOS or 4b/a” in combination with “mutation or polymorphism or variant” and in combination with “diabetic retinopathy or DR.” We also manually searched all the references of included studies to further identify additional relevant studies. Unpublished studies were not sought. For overlapping or republished studies, only the larger sample size or the most recent published papers were included in this meta-analysis. Publication language and publication date were not restricted in our search.

### 2.2. Inclusion and Exclusion Criteria

Studies included in this meta-analysis must meet the following criteria: (1) case-control or cohort studies; (2) studies evaluating the association between eNOS-4b/a polymorphism and DR risk; (3) subjects in the control group having type 2 diabetes but being free of DR; (4) human studies; and (5) having detailed data to calculate the odds ratio (OR) and 95% confidence interval (CI).

Studies were excluded if one of the following existed: (1) review articles or editorials; (2) case reports; (3) repeating or overlapping publications; (4) no report about the genotype frequency or insufficient information for data extraction.

### 2.3. Data Extraction

The following data were collected from each study: first author, publication date, region, ethnicity, sample size of cases and controls, genotype and allele frequencies of cases and controls, and genotyping methods. All the data were extracted independently by two investigators (Ze-jun Ma and Hui-Zhu Ren), according to the inclusion criteria above. Disagreements about eligibility were resolved through a discussion between the two investigators.

### 2.4. Statistical Analysis

In this meta-analysis, we evaluated the relationship between eNOS-4b/a polymorphism and DR risk using the allelic model (a versus b), the additive model (aa versus bb), the dominant model (aa + ab versus bb), and the recessive model (aa versus ab + bb) (see Figure 5). The alleles and genotypes between patients and control subjects were compared with OR and corresponding 95% CIs. A chi-square based* Q* statistics test and* I* ^2^ test were used to evaluate the heterogeneity between the studies (*P* < 0.10 and* I* ^2^ > 50% indicated the evidence of heterogeneity) [15]. The fixed effects model (FEM) was used when there was no statistical heterogeneity among the included studies; otherwise, the random effects model (REM) was used. Subgroup analysis was conducted according to different ethnicity. Sensitivity analysis was performed for estimating the stability of the meta-analysis. First, sensitivity analysis was carried out by exclusion of studies which failed the HWE test. Another analysis was done by omitting one study at a time to examine influence of one study on the overall summary estimate. Begg's funnel plot and Egger's test were carried out to assess possible publication bias [16, 17]. Asymmetric plot or the *P* value of Egger's test less than 0.05 suggested possible publication bias. Meta-analyses were performed using the statistical software Review Manager (version 5.2 for Windows, Cochrane Collaboration) and STATA software (version 12.0; Stata, College Station, TX), using two-sided *P* values.

## 3. Results

### 3.1. Characteristics of the Studies

**Figure 1:**
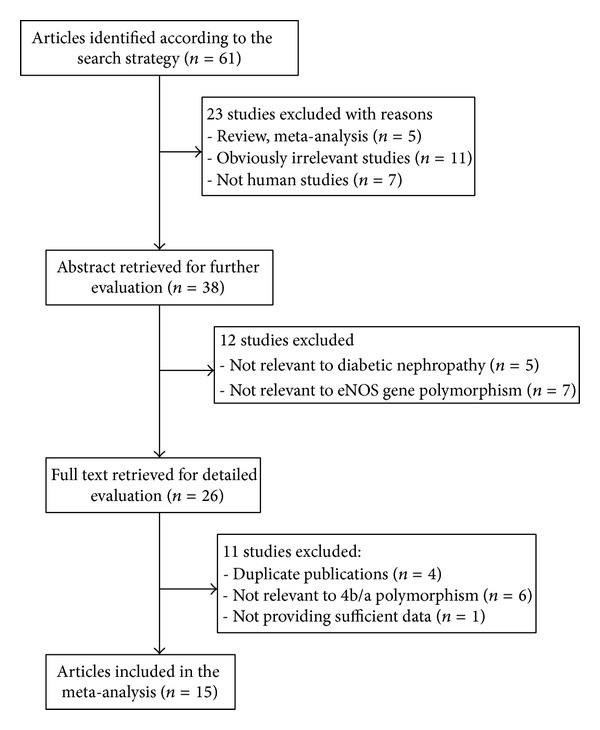
Flow chart of included studies.

Based on our search strategy, 61 potentially relevant articles were identified in Pubmed, Embase, CNKI, and Chinese Wanfang Database. A flow chart of study selection was shown in Figure 1. Of these, 46 were excluded because they did not meet the criteria or were overlapping publications. Finally, a total of 15 studies published between 2004 and 2012 met our inclusion criteria, involving 3,183 cases and 3,410 controls. The main characteristics of these studies were listed in Table 1. Of the ethnicity among all studies, four studies were performed on Caucasians [4, 13, 14, 18], ten were performed on Asian [12, 19–27], and one study was performed on West African [28]. The sample size in these studies varied considerably (ranging from 166 to 1446 individuals). The genotype and allele distributions for each study and HWE in controls were summarized in Table 2. Two genotyping methods were used to check genotypes in the studies including polymerase chain reaction (PCR) or PCR-restriction fragment length polymorphism (PCR-RFLP).

The distribution of the eNOS 4b/a genotype in control group was consistent with the HWE, except for two studies [23, 26]. Because excluding these two studies did not materially affect the results, they were still included in this analysis.

### 3.2. Meta-Analysis Results

**Figure 2:**
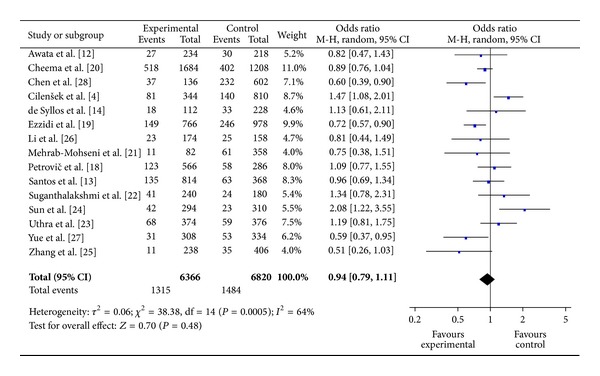
The forest plot of a versus b of eNOS polymorphism and overall DR risk.

**Figure 3:**
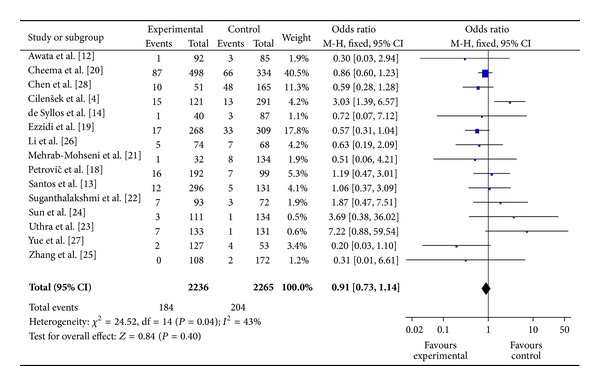
The forest plot of aa versus bb of eNOS polymorphism and overall DR risk.

**Figure 4:**
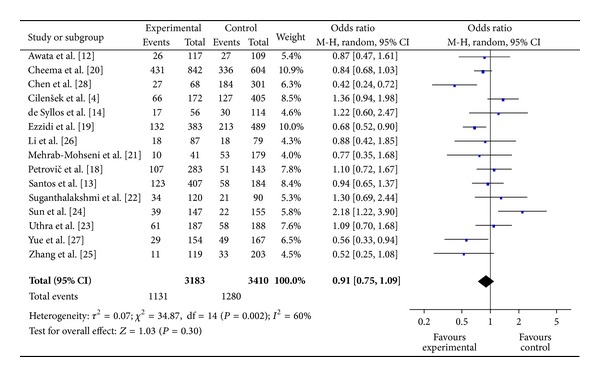
The forest plot of aa + ab versus bb of eNOS polymorphism and overall DR risk.

**Figure 5:**
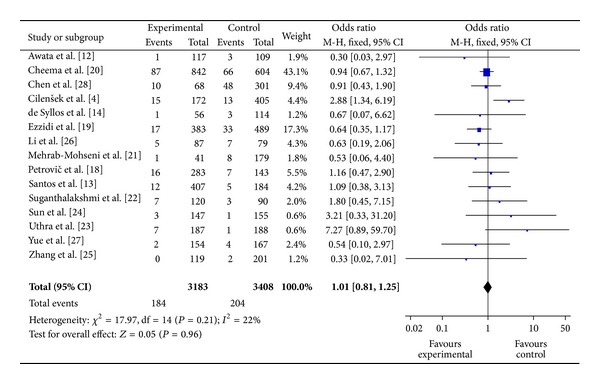
The forest plot of aa versus bb + ab of eNOS polymorphism and overall DR risk.

The main results of meta-analysis for eNOS 4b/a polymorphism with the risk of DR were shown in Table 3. No significant association between eNOS 4b/a polymorphism and susceptibility to DR was identified in any of the genetic models (allelic model: OR = 0.94, 95% CI: 0.79–1.11; additive model: OR = 0.91, 95% CI: 0.73–1.14; the recessive model: OR = 1.01, 95% CI: 0.81–1.25; and the dominant model: OR = 0.91, 95% CI: 0.75–1.09) (Figures 2, 3, and 4).

In the subgroup analysis by ethnicity, similarly, there was still no significant association detected in all genetic models among Asians and Caucasians. The results of the subgroup analyses were shown in Table 3.

### 3.3. Heterogeneity and Publication Bias

Some heterogeneity was found during the course of the study. Hence, the random effects model was used. Begg's funnel plot and Egger's test were performed to assess the publication bias of the eligible literatures in this meta-analysis. The shapes of the funnel plots in all genetic models did not reveal any evidence of obvious asymmetry. The Egger's test further confirmed the absence of publication bias for any of the four genetic models in this meta-analysis (*P* = 0.277 for dominant model, *P* = 0.313 for recessive model, *P* = 0.287 for additive model, and *P* = 0.764 for allelic model, resp.).

### 3.4. Sensitivity Analysis

Sensitivity analyses were performed to assess the stability of the results. Although two studies [23, 26] did not follow the HWE, the summary ORs were not materially altered including or excluding the studies (data shown in Table 3). Moreover, no other single study influenced the overall results (data not shown), which indicated that our results were statistically reliable and robust.

## 4. Discussion

The polymorphism of eNOS-4b/a gene has been associated with many vascular diseases including hypertension, diabetic retinopathy, and diabetic nephropathy in various populations [29, 30]. Variable results have been reported for the association of eNOS-4b/a polymorphism with DR [13, 20, 21]. In a systematic meta-analysis study [3], no relationship of the eNOS 4b/a polymorphism was found with DR development regardless of ethnicity. However, a recent meta-analysis study found that eNOS 4b/a has a protective effect against DR [31]. The present meta-analysis of 15 studies, including 3,183 cases and 3,410 controls, provided the most comprehensive analysis on the association of the eNOS 4b/a polymorphism with the risk of diabetic retinopathy. The results indicated that the eNOS 4b/a polymorphism was not associated with an increased risk of DR in the overall studied population. These findings were consistent with most of the studies that were included in our meta-analysis [13, 14, 23]. The lack of association between eNOS 4b/a polymorphism and diabetic retinopathy suggested that genetic variations in the eNOS 4b/a gene did not predict the risk of diabetic retinopathy in T2DM patients. It is possible that eNOS-derived NO plays a minor role in the development of diabetic retinopathy. In the subgroup analysis according to ethnicity, no significant association was observed in Asian and Caucasians in all genetic models. The results demonstrated that ethnic difference in genetic background and the living environment did not play an obvious role in the association between the eNOS 4b/a polymorphism and the risk of DR. Although the available genetic data do not implicate the eNOS 4b/a polymorphism as a determinant of DR susceptibility in Asian and Caucasians, further studies are needed to see if the eNOS 4b/a polymorphism can confer a risk of DR in other ethnic populations.

Publication bias is an important factor affecting us to get a reliable conclusion for meta-analysis. In this meta-analysis, no significant publication bias for 4b/a polymorphism in any of the above-mentioned inherited models was found, suggesting that the results observed should be stable. Sensitivity analyses did not significantly alter the results, also suggesting that our results were statistically reliable and stable.

Our study has several limitations that need to be taken into consideration when interpreting the results. First, significant between-study heterogeneity was detected in some comparisons and might distort the meta-analysis. Second, only published studies in English or Chinese were included for data analysis; some potential studies with other languages or unpublished could be missed. Third, this meta-analysis was based predominantly on Asian research. Only 4 studies involving Caucasians and one study involving Africans were included. No study from other parts of the world was found. This may develop a partial result. Fourth, our meta-analysis was based on unadjusted OR estimates. Despite these limitations, our study provides a better understanding of the association between eNOS-4b/a gene polymorphisms and risk of DR in type 2 diabetes.

In summary, this meta-analysis indicates that the eNOS 4b/a polymorphism is not associated with an increased risk of DR among type 2 diabetic subjects. Taking into account the limitations of this meta-analysis, further larger well-designed studies involving different ethnic populations, particularly referring to gene-gene and gene-environment interactions, are required to confirm this finding.

## Figures and Tables

**Table 1:** Main characteristics of included studies in the meta-analysis.

Author	Year	Country	Ethnicity	Case/control	Genotyping methods
Awata et al. [12]	2004	Japan	Asian	117/109	PCR
Cheema et al. [20]	2012	Indian	Asian	842/604	PCR-RFLP
Chen et al. [28]	2007	Ghana and Nigeria	West African	68/301	PCR
Cilenšek et al. [4]	2012	Slovenia	Caucasians	172/405	PCR
de Syllos et al. [14]	2006	Brazil	Caucasians	56/114	PCR
Ezzidi et al. [19]	2008	Bahrain	Asian	383/489	PCR-RFLP
Li et al. [26]	2010	china	Asian	87/79	PCR
Mehrab-Mohseni et al. [21]	2011	Iran	Asian	41/179	PCR
Petrovič et al. [18]	2008	Slovenia	Caucasians	283/143	PCR
Santos et al. [13]	2012	Brazil	Caucasians	407/184	PCR
Suganthalakshmi et al. [22]	2006	India	Asian	120/90	PCR-RFLP
Sun et al. [24]	2004	china	Asian	147/155	PCR-RFLP
Uthra et al. [23]	2007	India	Asian	187/188	PCR
Yue et al. [27]	1994	china	Asian	154/167	PCR

Zhang et al. [25]	2005	china	Asian	119/203	PCR

**Table 2:** The distribution of the 4b/a genotype and allele frequency for cases and controls.

Study	Distribution of 4b/a eNOS genotype						Allele frequency				HWE
	Cases			Controls			Cases		Controls		
	aa	ab	bb	aa	ab	bb	a	b	a	b	
Awata et al. [12]	1	25	91	3	24	82	27	207	30	188	Yes
Cheema et al. [20]	87	344	411	66	270	268	518	1166	402	806	Yes
Chen et al. [28]	10	17	41	48	136	117	37	99	232	370	Yes
Cilenšek et al. [4]	15	51	106	13	114	278	81	263	140	670	Yes
de Syllos et al. [14]	1	16	39	3	27	84	18	94	33	195	Yes
Ezzidi et al. [19]	17	115	251	33	180	276	149	617	246	732	Yes
Li et al. [26]	5	13	69	7	11	61	23	151	25	133	No
Mehrab-Mohseni et al. [21]	12	111	284	5	53	126	135	679	63	305	Yes
Petrovič et al. [18]	16	91	176	7	44	92	123	443	58	228	Yes
Santos et al. [13]	12	111	284	5	53	126	135	679	63	305	Yes
Suganthalakshmi et al. [22]	7	27	86	3	18	69	41	199	24	156	Yes
Sun et al. [24]	3	36	108	1	21	133	42	252	23	287	Yes
Uthra et al. [23]	7	54	126	1	57	130	68	306	59	317	No
Yue et al. [27]	2	27	125	4	45	118	31	277	53	281	Yes
Zhang et al. [25]	0	11	108	2	31	170	11	227	35	371	Yes

HWE: Hardy-Weinberg equilibrium, *P* < 0.05 was considered significant.

**Table 3:** Meta analysis of the association of eNOS-4b/a gene polymorphism with DR in type 2 diabetes.

Genetic model	Populations	Studies (*n*)	Number of cases/controls	Heterogeneity *Q* test *P*-value	*I* ^2^ (%)	OR (95% CI)	*P* value
a versus b	All	15	3183/3410	0.0005	64	0.94 (0.79–1.11) (REM)	0.48
	Asian	10	2265/2564	0.006	61	0.90 (0.73–1.11) (REM)	0.32
	Caucasians	4	918/846	0.30	18	1.17 (0.97–1.40) (FEM)	0.10
	HWE (yes)	13	2909/3143	0.0003	67	0.93 (0.77–1.12) (REM)	0.44

aa versus bb	All	15	3183/3410	0.04	43	0.91 (0.73–1.14) (FEM)	0.40
	Asian	10	2265/2564	0.21	24	0.78 (0.61–1.01) (FEM)	0.06
	Caucasians	4	918/846	0.26	25	1.64 (0.98–2.73) (FEM)	0.06
	HWE (yes)	13	2909/3143	0.06	41	0.88 (0.70–1.11) (FEM)	0.28

aa + ab versus bb	All	15	3183/3410	0.002	60	0.91 (0.75–1.09) (REM)	0.30
	Asian	10	2265/2564	0.0004	61	0.82 (0.65–1.04) (REM)	0.11
	Caucasians	4	918/846	0.58	0	1.13 (0.91–1.40) (FEM)	0.26
	HWE (yes)	13	2909/3143	0.0007	65	0.89 (0.72–1.10) (REM)	0.30

aa versus bb + ab	All	15	3183/3410	0.21	22	1.01 (0.81–1.25) (FEM)	0.96
	Asian	10	2265/2564	0.49	0	0.90 (0.70–1.15) (FEM)	0.39
	Caucasians	4	918/846	0.28	21	1.60 (0.97–2.65) (FEM)	0.07
	HWE (yes)	13	2909/3143	0.30	14	0.98 (0.78–1.23) (FEM)	0.88

OR: odds ratio; CI: confidence interval.

## References

[1] Marshall SM, Flyvbjerg A (2006). Prevention and early detection of vascular complications of diabetes. *British Medical Journal*.

[2] Yang W, Lu J, Weng J (2010). Prevalence of diabetes among men and women in China. *The New England Journal of Medicine*.

[3] Abhary S, Hewitt AW, Burdon KP, Craig JE (2009). A systematic meta-analysis of genetic association studies for diabetic retinopathy. *Diabetes*.

[4] Cilenšek I, Mankoč S, Petrovič MG, Petrovič D (2012). The 4a/4a genotype of the VNTR polymorphism for endothelial nitric oxide synthase (eNOS) gene predicts risk for proliferative diabetic retinopathy in Slovenian patients (Caucasians) with type 2 diabetes mellitus. *Molecular Biology Reports*.

[5] Churchill AJ, Carter JG, Ramsden C (2008). VEGF polymorphisms are associated with severity of diabetic retinopathy. *Investigative Ophthalmology and Visual Science*.

[6] Niu W, Qi Y (2012). An updated meta-analysis of methylenetetrahydrofolate reductase gene 677C/T polymorphism with diabetic nephropathy and diabetic retinopathy. *Diabetes Research and Clinical Practice*.

[7] Bellini MH, Figueira MN, Piccoli MF (2007). Association of endothelial nitric oxide synthase gene intron 4 polymorphism with end-stage renal disease. *Nephrology*.

[8] Moncada S, Higgs A (1993). The L-arginine-nitric oxide pathway. *The New England Journal of Medicine*.

[9] Toda N, Nakanishi-Toda M (2007). Nitric oxide: ocular blood flow, glaucoma, and diabetic retinopathy. *Progress in Retinal and Eye Research*.

[10] Li Q, Verma A, Han P-Y (2010). Diabetic eNOS-knockout mice develop accelerated retinopathy. *Investigative Ophthalmology and Visual Science*.

[11] Rao AA, Thota H, Gumpeny RS (2008). Bioinformatics analysis of diabetic retinopathy using functional protein sequences. *Medical Hypotheses*.

[12] Awata T, Neda T, Iizuka H (2004). Endothelial nitric oxide synthase gene is associated with diabetic macular edema in type 2 diabetes. *Diabetes Care*.

[13] Santos KG, Crispim D, Canani LH, Ferrugem PT, Gross JL, Roisenberg I (2012). Relationship of endothelial nitric oxide synthase (eNOS) gene polymorphisms with diabetic retinopathy in Caucasians with type 2 diabetes. *Ophthalmic Genetics*.

[14] de Syllos RWC, Sandrim VC, Lisboa HRK, Tres GS, Tanus-Santos JE (2006). Endothelial nitric oxide synthase genotype and haplotype are not associated with diabetic retinopathy in diabetes type 2 patients. *Nitric Oxide: Biology and Chemistry*.

[15] DerSimonian R, Laird N (1986). Meta-analysis in clinical trials. *Controlled Clinical Trials*.

[16] Begg CB, Mazumdar M (1994). Operating characteristics of a rank correlation test for publication bias. *Biometrics*.

[17] Egger M, Smith GD, Schneider M, Minder C (1997). Bias in meta-analysis detected by a simple, graphical test. *British Medical Journal*.

[18] Petrovič MG, Cilenšek I, Petrovič D (2008). Manganese superoxide dismutase gene polymorphism (V16A) is associated with diabetic retinopathy in Slovene (Caucasians) type 2 diabetes patients. *Disease Markers*.

[19] Ezzidi I, Mtiraoui N, Mohamed MBH, Mahjoub T, Kacem M, Almawi WY (2008). Endothelial nitric oxide synthase Glu298Asp, 4b/a, and T-786C polymorphisms in type 2 diabetic retinopathy. *Clinical Endocrinology*.

[20] Cheema BS, kohli HS, Sharma R, Bhansali A, Khullar M (2012). Endothelial nitric oxide synthase gene polymorphism and type 2 diabetic retinopathy among Asian Indians. *Acta Diabetologica*.

[21] Mehrab-Mohseni M, Tabatabaei-Malazy O, Hasani-Ranjbar S (2011). Endothelial nitric oxide synthase VNTR (intron 4 a/b) polymorphism association with type 2 diabetes and its chronic complications. *Diabetes Research and Clinical Practice*.

[22] Suganthalakshmi B, Anand R, Kim R (2006). Association of VEGF and eNOS gene polymorphisms in type 2 diabetic retinopathy. *Molecular Vision*.

[23] Uthra S, Raman R, Mukesh BN (2007). Intron 4 VNTR of endothelial nitric oxide synthase (eNOS) gene and diabetic retinopathy in type 2 patients in southern India. *Ophthalmic Genetics*.

[24] Sun H, Yang M, Liu S (2004). Association of endothelial nitric oxide synthase gene polymorphisms with type 2 diabetes meatus and diabetic retinopath. *Chinese Journal of Endocrinology and Metabolism*.

[25] Zhang M, Liu H, Xu Y, Wang Y, Song D, Li H (2005). Endothelial nitric oxide synthase gene intron 4 polynlorphism is associated with type 2 diabetic retinopathy. *Chinese Journal of Endocrinology and Metabolism*.

[26] Li M, Li X, Zhao M, Ji L (2010). Association of genetic polymorphism of nitric oxide synthase and diabetic retinopathy. *Chinese Journal of Ocular Fundus Diseases*.

[27] Yue M, Yu X, Dai H, Long L (1994). Diabetic retinopathy and variable number tandem repeat polymorphism in intron 4 of endothelial nitric oxide synthase gene. *Biochemical and Biophysical Research Communications*.

[28] Chen Y, Huang H, Zhou J (2007). Polymorphism of the endothelial nitric oxide synthase gene is associated with diabetic retinopathy in a cohort of West Africans. *Molecular Vision*.

[29] Nadaud S, Bonnardeaux A, Lathrop M, Soubrier F (1994). Gene structure, polymorphism and mapping of the human endothelial nitric oxide synthase gene. *Biochemical and Biophysical Research Communications*.

[30] Wang XL, Wang J (2000). Endothelial nitric oxide synthase gene sequence variations and vascular disease. *Molecular Genetics and Metabolism*.

[31] Marshall SM, Flyvbjerg A (2006). Prevention and early detection of vascular complications of diabetes. *British Medical Journal*.

